# New Method of Dressing Wounds and Ulcers

**Published:** 1845-04

**Authors:** 


*New Method of Dressing Wounds and Ulcers.*---By Dr. Laugier.---This method consists in applying, on the surface of the wound or ulcer, a solution of gum-arabic, and on it a bit of gold-beater's skin; thus dressed, a wound, an inch in diameter, was reduced in the space of eight days to one-third or one-sixth of an inch in extent. Cicatrization took place so rapidly, that the granulations, covered with a thin epidermis, were as numerous and visible as before, but could be touched without causing pain. A wound, produced by amputation of the breast, highly inflamed, about four and a half inches in length, and one and a half inches in breadth, under this treatment, healed rapidly, and purulent secretion did not take place. The author proposes applying this method to a wound left by amputation of a thigh.---*Med. Times.*---*Medical News.*

